# Phytocannabinoid-rich galenic preparations for topical administration: extraction and stability testing

**DOI:** 10.3389/fphar.2023.1230728

**Published:** 2023-08-01

**Authors:** Dominika Kaczorová, Jaroslav Peč, Tibor Béres, Nikola Štefelová, Sanja Ćavar Zeljković, Václav Trojan, Anežka Kosmáková Janatová, Pavel Klouček, Petr Tarkowski

**Affiliations:** ^1^ Czech Advanced Technology and Research Institute, Palacký University, Olomouc, Czechia; ^2^ Department of Genetic Resources for Vegetables, Medicinal and Special Plants, Centre of the Region Haná for Biotechnological and Agricultural Research, Crop Research Institute, Olomouc, Czechia; ^3^ Department of Biochemistry, Faculty of Science, Palacký University, Olomouc, Czechia; ^4^ Pharm & Herb s. r. o, Prostějov, Czechia; ^5^ Cannabis Facility, Centre for Translational Medicine, International Clinical Research Centre, St. Anne’s University Hospital, Brno, Czechia; ^6^ Department of Food Science, Faculty of Agrobiology, Food and Natural Resources, Czech University of Life Sciences Prague, Prague, Czechia

**Keywords:** *Cannabis sativa* L., medical cannabis, phytocannabinoid, galenic preparation, extract, stability, dermatology, topical application

## Abstract

Although medical cannabis was legalized in Czechia in 2013 and its use in topical treatments of skin disorders is now allowed, galenic formulations prepared from medical cannabis have not been widely implemented in the Czech healthcare system. One of the main reasons is the lack of a straightforward standardized protocol for their preparation. Cannabinoids, e.g., cannabidiol (CBD) and tetrahydrocannabinol (THC), have been shown to have therapeutic effects on various skin conditions, such as atopic dermatitis, psoriasis, scleroderma, acne and skin pigmentation. Recognizing the potential of dermatological treatment with medical cannabis, the present study aimed to evaluate the extraction capacity of various pharmaceutical bases for cannabinoids and the stability of prepared galenic formulations for dermatological applications with respect to cannabinoid content. The results showed that the stability of cannabinoids in formulations depended on the bases’ physical and chemical properties. The highest THC decomposition was observed in cream bases and Vaseline, with estimated percentage loss of total content of up to 5.4% and 5.6% per week, respectively. In contrast, CBD was more stable than THC. Overall, the tested bases were comparably effective in extracting cannabinoids from plant material. However, olive oil and Synderman bases exhibited the highest cannabinoid extraction efficiencies (approximately 70%) and the best storage stabilities in terms of the content of monitored compounds. The proposed preparation protocol is fast and easily implementable in pharmacies and medical facilities.

## 1 Introduction

Legislation on *Cannabis sativa* L. in Czechia has undergone major changes in the last 10 years, starting with an amendment to the law on addictive substances in 2013 (Parliament of the Czech Republic, 2013), which enabled the use of medical cannabis for treatment and research. It was followed by the release of an implementing decree in 2015 (Ministry of Health and Ministry of Agriculture, 2015), which stated rules for the prescription, preparation, distribution, dispensing and use of cannabis for medical purposes, listed specialist physicians allowed to prescribe medical cannabis electronically and made pharmacies responsible for dispensing the drug. Another important change came at the beginning of 2020 when the law stipulated that 90% of the cost of medical cannabis dispensed in pharmacies would be covered by public health insurance up to 30 g per month. Recently, Czechia has joined Switzerland in setting a 1% legal limit on *trans*-∆^9^-tetrahydrocannabinol (*trans*-∆^9^-THC) in the plant and products (Parliament of the Czech Republic, 2021). In contrast, the *trans*-∆^9^-THC content limit is generally set at 0.2% in the European Union (EU), except for Italy, which has a 0.6% *trans*-∆^9^-THC limit. Moreover, the novelization has also enabled the use of cannabis extract (Ministry of Health and Ministry of Agriculture, 2022). However, despite these developments, it is still not a commonly accessible medical product.

Above all, the legislation on medical cannabis administration differs among EU member states, preventing common agreement on its distribution. Some European countries have even published official pharmacopeial monographs dedicated to standardized preparation and evaluation of medical cannabis or extracts on a state basis (Dutch Office for Medicinal Cannabis, 2014; German Pharmacopoeia, 2017; Danish Cannabis Monograph, 2019; Swiss Cannabis Monograph, 2019). In this context, medical cannabis quality is regulated and controlled by state agencies, even though a harmonized EU cannabis monograph is expected to be published (Official Danish Cannabis-Monograph, 2019).

The endocannabinoid system is involved in maintaining homeostasis, as well as skin barrier function and its regenerative capacity. The cannabinoid receptors CB_1_ and CB_2_ have been identified in various cells of skin and hair follicles. Numerous skin disorders, such as atopic dermatitis, psoriasis, scleroderma, acne, hair growth disorders, skin pigmentation, allergic contact dermatitis and diseases related to keratin formation, are associated with dysregulation of the endocannabinoid system (Río et al., 2018; Sheriff et al., 2020; Peč et al., 2022). In a recent study, CBD showed a favorable response in the topical treatment of psoriasis (Puaratanaarunkon et al., 2022). Nevertheless, the pharmacodynamics of the cannabinoid mechanism of action is extremely complex and may include components of other signaling cascades, e.g., transient receptor potential vanilloid (TRPV) channels, peroxisome proliferator-activated receptors (PPARs), orphan G protein-coupled receptors (GPCRs), other non-cannabinoid receptors (serotonin, adenosine, glycine) and enzymatic systems involved in the biosynthesis and degradation of endocannabinoids (cyclooxygenases, lipoxygenases) (Kupczyk et al., 2009; Jeong et al., 2019; Proksch et al., 2019; Tóth et al., 2019; Peč et al., 2022). Furthermore, transdermal application of cannabinoids could be used not only for topical disorders but also as an alternative to cannabinoid administration into the systemic bloodstream. Recently, the first human pharmacokinetic study investigating the bioavailability of cannabidiol (CBD) and *trans*-∆^9^-THC was published. Using the reported transdermal technology, the cannabinoids successfully penetrated the human skin and entered the systemic circulation, with the conclusion that the application was safe and well tolerated by participants (Varadi et al., 2023).

In Czechia, ethanolic cannabis tinctures were applied to patients at the Olomouc University Hospital in the 1950s in relation to the research at the Palacký University Olomouc by Professors Kabelík, Šantavý and Krejčí (Peč et al., 2022). However, usually, topical (local skin effect) and/or transdermal (systemic effect) application of cannabis extracts (ointments) involves patients self-medicating using home-made products. Regarding dermatological applications, the topical treatment of dermatoses and mucosal lesions is formulated in the Czech implementing decree (Ministry of Health and Ministry of Agriculture, 2015) as one of the possible variants of medical cannabis administration. However, so far, it has not been practically implemented in the Czech healthcare system. One of the main reasons is the lack of a standardized technological procedure for the preparation of dermatological dosage forms in pharmacies. Another aspect is the absence of relevant and detailed studies of the extraction efficiency of pharmaceutically important cannabis constituents into ointment-based vehicles.

Recognizing the potential of dermatological treatment with medical cannabis, the goal of this study was to design a technological protocol for the preparation of galenic cannabis-based extracts implementable in pharmacy settings. Therefore, only materials and equipment commonly available in pharmacies were used for their preparation. Several pharmaceutical vehicles are available for manufacturing topical products with various physical and chemical characteristics (Table 1), e.g., liquid or semisolid, hydrophobic or hydrophilic. In this work, we demonstrated the extractive capabilities of different pharmaceutical ointment bases (POBs) intended for the preparation of dermatological dosage forms under standardized conditions in a pharmacy care facility. To the best of our knowledge, this is the first study to test the extractability of various POBs for cannabis. The findings could help pharmacies and the medical profession to provide dermatological treatments for patients, which are in high demand.

**TABLE 1:** Types of pharmaceutical ointment bases (vehicles).

Base type	Characteristics	Examples
Ointments (unguenta)	Hydrophobic	Petrolatum (*vaselinum album*/*flavum*)
	Wax (w/o)	Synderman (SydoFarm^®^)
	Hydrophilic	Macrogoli *unguentum*
Creams (cremores)	Oleocreams (w/o)	Cremor/*unguentum leniens*
	Hydrocreams (o/w)	Ambiderman (AmiFarm^®^)
		Cremor neoaquasorb
		Neoaquasorb (AquaNeoFarm^®^) *unguentum*
		Pentravan^®^
Gels (gelata)	Hydrogels	Carbomer gel
	Oleogels	
Liquids	Oils	Olive oil (*olivae oleum*)
		Castor oil
		Liquid paraffin (mineral oil)
	Water	Purified Water
	Water-soluble	Polyethylene glycol (PEG)
	Alcohols	Ethanol
		Isopropanol

w/o … water-in-oil type of emulsion.

o/w … oil-in-water type of emulsion.

## 2 Materials and methods

### 2.1 Materials

Dried cannabis inflorescences of chemotype I (high-THC) provided by the Czech University of Life Sciences, Prague were used for assessing the extraction capacity of POBs. A second batch of cannabis was used for the stability testing and consisted of chemotype I inflorescences obtained from St. Anne´s University Hospital, Brno, as well as chemotype II inflorescences (THC + CBD) supplied by Elkoplast Slušovice, s.r.o. The chemotype III (high-CBD) plants were cultivated, harvested and dried at the Crop Research Institute in Olomouc.

POBs—Vaseline (*vaselinum album*), Synderman (SydoFarm^®^), Ambiderman (AmiFarm^®^), Cremor neoaquasorb (AquaNeoFarm^®^ Cremor), Neoaquasorb (AquaNeoFarm^®^) *unguentum*, Pentravan^®^, olive oil (*Olivae oleum*), paraffin (*paraffinum liquidum*), propylene glycol, ricin oil were purchased from the Pharmacy of the Olomouc University Hospital (manufacturer: Fagron, Czechia).

Stock solutions of pure certified analytical standards (Cerilliant^®^) of cannabigerol (CBG), cannabigerolic acid (CBGA), cannabinol (CBN), cannabinolic acid (CBNA), ∆^8^-tetrahydrocannabinol (∆^8^-THC), cannabichromene (CBC), cannabichromenic acid (CBCA), ∆^9^-tetrahydrocannabivarin (∆^9^-THCV), ∆^9^-tetrahydrocannabivarinic acid (∆^9^-THCVA), cannabidivarin (CBDV), cannabidivarinic acid (CBDVA), cannabicyclol (CBL) and cannabicyclolic acid (CBLA) were purchased from Merck (Darmstadt, Germany). Standards of (-)-*trans*-∆^9^-tetrahydrocannabinol ((-)-*trans*-∆^9^-THC), (-)-*trans*-∆^9^-tetrahydrocannabinolic acid ((-)-*trans*-∆^9^-THCA-A), cannabidiol (CBD) and cannabidiolic acid (CBDA) were purchased from Lipomed (Arlesheim, Switzerland). Other solvents and chemicals were from the following manufacturers: formic acid, Supelco^®^ LC-MS grade water, LiChrosolv^®^ 2-propanol (Merck, Darmstadt, Germany), HPLC grade acetonitrile (Fisher Chemicals, Hampton, United States), 96% ethanol (Lach:ner, Neratovice, Czechia).

### 2.2 Decarboxylation

Dried cannabis inflorescences, stripped of leaves and larger stems were heated in closed glass bottles in a hot-air dryer at 121°C for 30 min according to conditions for preparing medical cannabis recommended by Landa and Jurica. (2020).

In the case of the chemotype III material, two 30 min heating were applied (total heating of 60 min) owing to incomplete decarboxylation of CBDA to CBD.

### 2.3 Phytocannabinoid analysis

Cannabinoids were analyzed by ultra-high performance liquid chromatography coupled to a UV detector (UHPLC-UV) using an UltiMate™ 3000 UHPLC system (Thermo Fisher Scientific, Waltham, MA, United States). Plant and extract samples were prepared according to a previously reported methodology (Béres et al., 2019) with some modifications. Briefly, 45 ± 5 mg of each replicate was weighed, 1.8 mL of 96% ethanol was added and the samples were sonicated for 30 min at laboratory temperature (60°C for *vaselinum album* and SydoFarm^®^ extracts), as recommended in Ciolino et al. (2018). After 10 min centrifugation (21 200 × g, laboratory temperature), the supernatant was diluted with 70% acetonitrile (ACN) containing 0.1% formic acid and filtered through CHS FilterPure filters (nylon filters, diameter 13 mm, porosity 0.22 μm; Chromservis, Prague, Czechia).

Two different methods were used for the analysis. Method I was used for the analysis of ointment extracts and utilized chromatographic conditions detailed in the Dutch Office for Medicinal Cannabis (OMC) monograph (Dutch Office for Medicinal Cannabis, 2014). Separation was performed on a Waters Acquity C18 (150 × 2.1 mm; 1.7 µm particle size) column (Waters Corp., Milford, MA, United States) kept at 30°C. The mobile phase comprised water (A) and acetonitrile (B), both containing 0.1% (v/v) of formic acid. A binary gradient started at 70% B, held for 6 min, then increased to 100% B during 4.5 min and held for 0.2 min. Afterwards, the proportion of B was decreased to 70% during 0.3 min. Finally, the column was re-equilibrated at the initial conditions for 1.5 min. The flow rate was 0.4 mL/min and the injection volume was 10 µL. A wavelength of 228 nm was used for detection and Xcalibur 1.2 software (Thermo Fisher Scientific) was used for data processing. For quantification, calibration solutions of 17 phytocannabinoid standards were measured.

**FIGURE 1:**
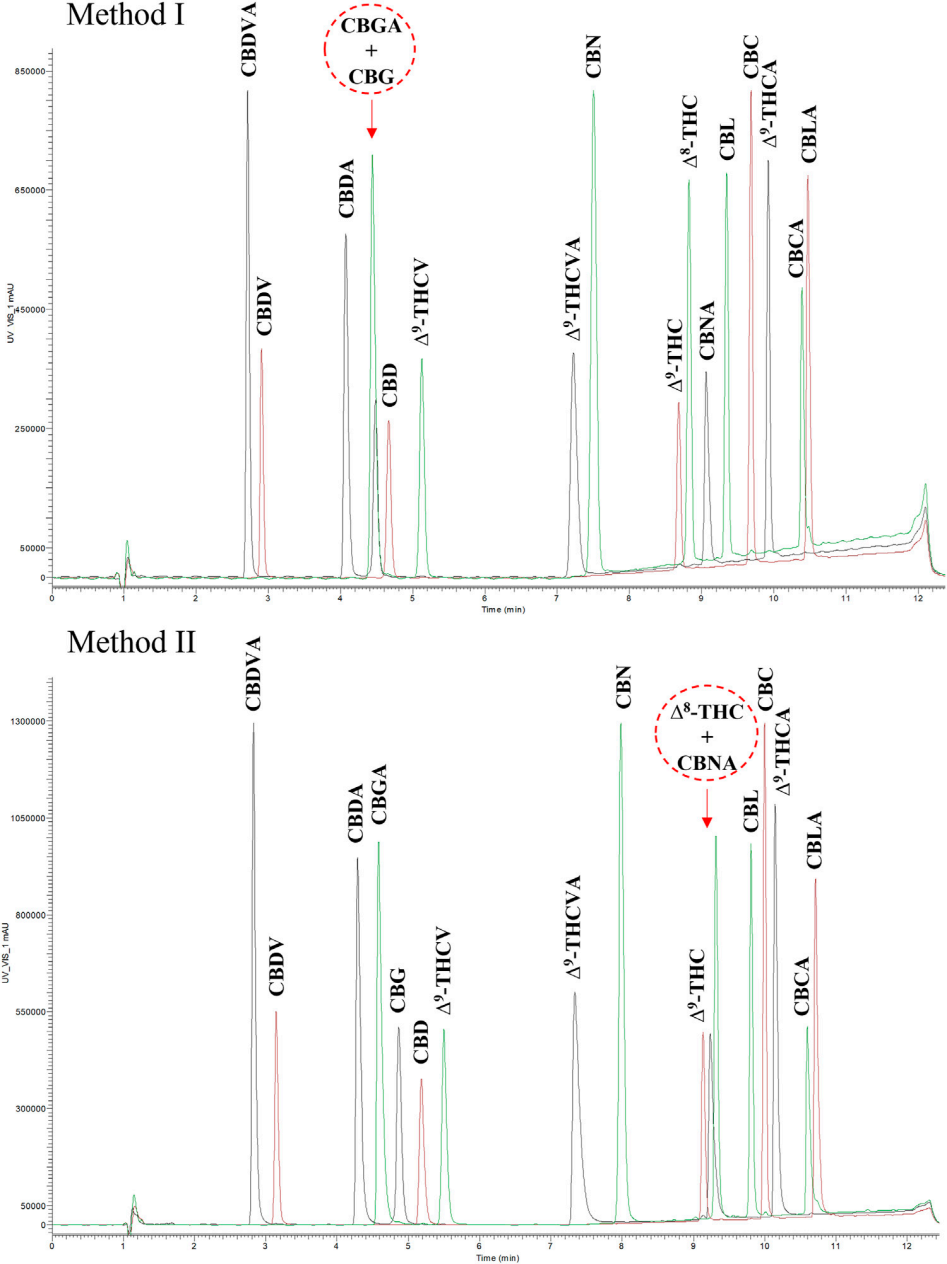
Chromatographic analysis of 17 cannabinoid standards by two different UHPLC-UV methods used in this work. Coeluting analytes are highlighted in red.∆^9^-THC, (-)-*trans*-∆^9^-tetrahydrocannabinol; ∆^9^-THCA, (-)-*trans*-∆^9^-tetrahydrocannabinolic acid; CBD, cannabidiol; CBDA, cannabidiolic acid; CBG, cannabigerol; CBGA, cannabigerolic acid; CBN, cannabinol; CBNA, cannabinolic acid; CBC, cannabichromene; CBCA, cannabichromenic acid; CBDV, cannabidivarin; CBDVA, cannabidivarinic acid; ∆^9^-THCV, ∆^9^-tetrahydrocannabivarin; ∆^9^-THCVA, ∆^9^-tetrahydrocannabivarinic acid; ∆^8^-THC, ∆^8^-tetrahydrocannabinol, CBL, cannabicyclol; CBLA, cannabicyclolic acid.

Another UHPLC-UV method (Method II) was used for the analysis of plant material (inflorescences) to avoid the coelution of CBGA and CBG in Method I (Figure 1). The chromatographic conditions used in Method II were based on Wang et al. (2018) as recommended by the United States Pharmacopeia (USP) Cannabis Expert Panel (Sarma et al., 2020). Separation was performed on a Waters Cortecs UPLC C18 (100 mm × 2.1 mm, 1.6 μm particle size) column (Waters Corp., Milford, MA, United States) kept at 35°C. The mobile phase comprised water (A) and acetonitrile (B), both containing 0.05% (v/v) formic acid. The flow rate was 0.3 mL/min. The binary gradient, injection volume and detection wavelength were the same as for Method I described above.

### 2.4 Extract preparation and stability test

Cannabis ointment extracts were prepared by the following procedure. An adequate amount of non- or decarboxylated cannabis inflorescence was homogenized in a mortar bowl with a pestle, then 1.5 g of the homogenized material was mixed with 30 g of POB in a 100 mL glass beaker to prepare a 5% cannabis extract (w/w). The mixtures were placed under a pharmaceutical infrared lamp (Heros, infrared bulb Philips 250 W, 230 V BR125) and heated for 30 min with occasional stirring. Afterwards, they were hot filtered through cotton gauze into plastic centrifuge tubes, and the weight was adjusted to 30 g after cooling to the laboratory temperature. For extraction time optimization, sampling was performed after 30, 60, 90, and 120 min of extraction under the infra lamp heating.

Regarding alcohol extraction testing, the samples were prepared by mixing 1.5 g of decarboxylated cannabis with 30 mL of 96% ethanol or isopropanol in plastic centrifuge tube. Then, the samples were macerated or sonicated in the ultrasonic bath for 30 min at laboratory temperature. After filtration, the volume was not adjusted.

For the time stability test, six POBs with different physical and chemical properties were selected as follows:

(a) *Vaselinum album*—hydrophobic ointment; (b) SydoFarm^®^ - w/o wax ointment (Synderman-type ointment); (c) AquaNeoFarm^®^ *unguentum*—anhydrous neoaquasorb, with water creates o/w hydro cream; (d) AmiFarm^®^ - o/w hydro-cream with an aqueous phase (Ambiderman cream); (e) *olivae oleum*—liquid olive oil of pharmaceutical grade; (f) Pentravan^®^—‘penetration enhancing vanishing cream’—a vehiculum suitable for transdermal drug delivery.

Each prepared ointment was separated into two aliquots (each 15 g) in plastic tubes and stored at laboratory temperature or in the refrigerator (at 7°C). Three replicates of each ointment type were prepared. The extracts were acclimatized to the laboratory temperature and thoroughly stirred before sampling for the phytochemical analysis. The monitoring period was set at 12 weeks. Sampling was performed every second week during the first phase of the storage period (6 weeks) and every third week during the second phase.

### 2.5 Statistical analysis

Statistical analysis was performed in RStudio (R Software version 4.1.0). Regression analysis was carried out to determine whether there was a significant decrease over time in the cannabinoid content (under the given conditions). A separate analysis was conducted for each chemotype. Regression models with cannabinoid content (in log-scale) set as the response variable and week (continuous variable), base (categorical variable with 6 categories), temperature (categorical variable with 2 categories) and interactions between them set as explanatory variables were considered. The estimated regression parameters were used to estimate the relative percentage change in the cannabinoid content after 1 week (for each base and temperature). The change was considered significant (at a statistical significance level α = 0.01) if the respective 99% confidence interval did not cover zero.

## 3 Results

### 3.1 Decarboxylation

Decarboxylation of the cannabis material was performed in closed vessels at 121°C for 30 min in a hot-air dryer as per conditions set in Czech medical practice. The original (before decarboxylation) and final (after decarboxylation) measured concentrations of phytocannabinoids are shown in Table 2. Phytochemical analysis was performed using the UHPLC-UV method described above.

**TABLE 2:** Phytocannabinoid content (%, w/w) in three different chemotypes (I, II, III) of *Cannabis sativa* L. before and after decarboxylation. Mean ± SD (*n* = 3).

	Dried (non-decarboxylated)% (w/w)			Decarboxylated% (w/w)		
Analyte	Chemotype I	Chemotype II	Chemotype III	Chemotype I	Chemotype II	Chemotype III
∆^9^-THC	0.59 ± 0.06	0.87 ± 0.04	0.16 ± 0.00	5.87 ± 0.23	4.07 ± 0.20	0.44 ± 0.01
∆^9^-THCA	5.67 ± 0.32	4.88 ± 0.09	0.35 ± 0.00	0.78 ± 0.07	0.85 ± 0.10	< LLOQ
CBD	< LLOQ	0.59 ± 0.02	1.86 ± 0.04	< LLOQ	4.42 ± 0.24	9.65 ± 0.34
CBDA	< LLOQ	9.74 ± 0.15	11.45 ± 0.10	< LLOQ	5.57 ± 0.17	2.05 ± 0.03
CBG	0.03 ± 0.00	0.10 ± 0.01	0.04 ± 0.00	0.25 ± 0.02	0.17 ± 0.01	0.08 ± 0.01
CBGA	0.26 ± 0.01	0.34 ± 0.03	0.15 ± 0.00	0.19 ± 0.02	0.17 ± 0.02	< LLOQ
CBN	< LLOQ	< LLOQ	< LLOQ	< LLOQ	0.07 ± 0.00	< LLOQ
CBNA	< LLOQ	0.11 ± 0.00	< LLOQ	< LLOQ	0.06 ± 0.00	< LLOQ
CBC	< LLOQ	< LLOQ	0.09 ± 0.01	0.04 ± 0.00	0.30 ± 0.02	0.46 ± 0.01
CBCA	0.10 ± 0.01	0.51 ± 0.02	0.46 ± 0.01	0.04 ± 0.00	0.27 ± 0.01	0.06 ± 0.00
CBDV	< LLOQ	< LLOQ	< LLOQ	< LLOQ	< LLOQ	0.18 ± 0.01
CBDVA	< LLOQ	0.04 ± 0.00	0.21 ± 0.00	< LLOQ	< LLOQ	0.03 ± 0.00

LLOQ, lower limit of quantification (0.025%); ∆^9^-THC, (-)-*trans*-∆^9^-tetrahydrocannabinol; ∆^9^-THCA, (-)-*trans*-∆^9^-tetrahydrocannabinolic acid; CBD, cannabidiol; CBDA, cannabidiolic acid; CBG, cannabigerol; CBGA, cannabigerolic acid; CBN, cannabinol; CBNA, cannabinolic acid; CBC, cannabichromene; CBCA, cannabichromenic acid; CBDV, cannabidivarin; CBDVA, cannabidivarinic acid. The concentrations of ∆^9^-tetrahydrocannabivarin, ∆^9^-tetrahydrocannabivarinic acid, ∆^8^-tetrahydrocannabinol, cannabicyclol and cannabicyclolic acid were below the LLOQ, value.

**FIGURE 2:**
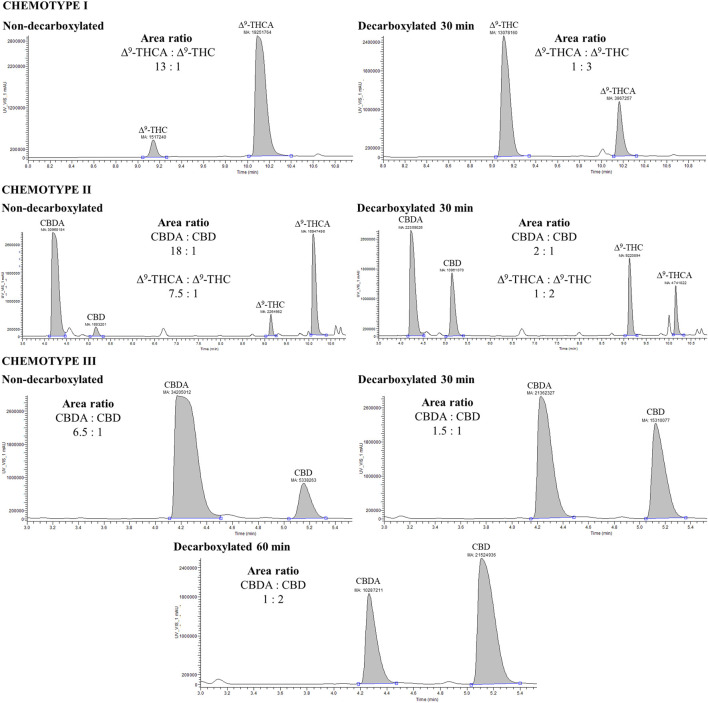
UHPLC-UV chromatograms of cannabinoid analysis of three different cannabis chemotypes (I, II, III) before and after decarboxylation. The comparison of the area ratios is only relative because of the saturated signal of highly concentrated analytes.

In the case of chemotype III, the procedure was repeated due to incomplete decarboxylation of CBDA to CBD. After the first decarboxylation (30 min), more than 60% of CBDA was still in acidic form. The same plant material was heated again (total time 1 h), enabling approximately 80% of the theoretical yield of CBD (calculated from the equation: initial content of CBDA * 0.877) to be reached. Although the time and temperature were initially insufficient for CBDA decarboxylation of the chemotype II variety, they were appropriate for ∆^9^-THCA decarboxylation within the same material (Figure 2). The initial ∆^9^-THCA concentration of 4.88% ± 0.09% decreased to 0.85% ± 0.10% (w/w) after 30 min decarboxylation, and the theoretical yield of decarboxylated ∆^9^-THC reached 80%. In contrast, the CBDA content decreased from 9.74% ± 0.15% to 5.57% ± 0.17% (w/w), leaving more than half of CBD in the acidic form. This may be due to the higher thermal stability of CBDA compared to ∆^9^-THCA, which has a higher decarboxylation rate constant (Wang et al., 2016; Moreno et al., 2020). In addition, the high content of CBDA in chemotype II (twice the ∆^9^-THCA content) may have affected the decarboxylation kinetics. On the other hand, the decarboxylation conditions resulted in the 80% calculated yield of ∆^9^-THC in chemotype I, corresponding to reduction of the ∆^9^-THCA content from 5.67% ± 0.32% to 0.78% ± 0.07% (w/w). Prolonged decarboxylation may result in a higher content of CBD, as observed for the chemotype III sample. However, ∆^9^-THC may be oxidized to CBN during long-time heating, which should be avoided because of the 1% CBN content limit in medical cannabis (Ministry of Health and Ministry of Agriculture, 2015).

### 3.2 Extraction capacity assessment

Assessment of the extraction capacity included selection of the most appropriate POB and extraction time optimization. Re-extraction of the plant material was also considered. The specified amount of decarboxylated and homogenized flowers of the *Cannabis sativa* chemotype I were mixed with POBs to prepare a 5% extract. The tested POBs showed different extractive capabilities for cannabinoids (Table 3, represented by the prevalent cannabinoid in the extracted material, ∆^9^-THC). The ∆^9^-THC extraction efficiency (EE, %) was calculated as follows:

$$EE\;\left(\%\right)=\frac{Experimental\;THC\;content}{Theoretical\;THC\;content}\times 100$$

**TABLE 3:** Extraction efficiency (%) of ∆^9^-THC in tested pharmaceutical ointment bases (POBs). 5% extracts (w/w) were prepared from decarboxylated and homogenized cannabis inflorescences of chemotype I.

POBs	Experimental ∆^9^-THC content (%, w/w)	Theoretical maximal ∆^9^-THC content (%, w/w)	Extraction efficiency (%)
*Olivae oleum*	0.225	0.321	70
*Vaselinum album*	0.160	0.321	50
Propylene glycol	0.208	0.302	69
Ricin oil	0.153	0.302	50
*Paraffinum liquidum*	0.161	0.302	53
Cremor neoaquasorb	0.160	0.302	53
Synderman	0.204	0.302	67
Ambiderman	0.098	0.321	31
Neoaquasorb *unguentum*	0.114	0.321	36

The theoretical maximal THC content in each 5% extract (%, w/w) was calculated according to the following equation:

$$Maximal\;THC\;\left(\%\right)=measured\;THC\;content\;in\;the\;plant\;material\;\left(\%\right)\times 0.05$$

Pharmaceutical-grade olive oil, propylene glycol and Synderman showed the highest extractive capabilities. On the other hand, Ambiderman and Neoaquasorb *unguentum* extracted the smallest quantity of ∆^9^-THC.

**FIGURE 3:**
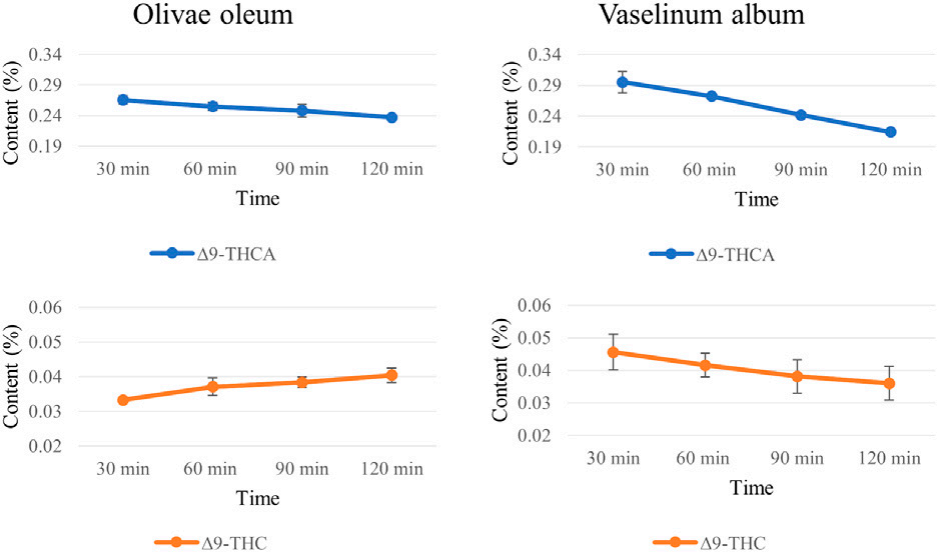
∆^9^-THCA and ∆^9^-THC extraction (%, w/w) into olive oil **(A)** and white Vaseline **(B)** during the monitored extraction time. The cannabinoids were extracted from non-decarboxylated chemotype I inflorescences. Mean ± SD (*n* = 3).

The time required for optimal extraction of cannabinoids from non-decarboxylated chemotype I cannabis inflorescences was tested for *olivae oleum* and *vaselinum album*. Sampling was performed after 30, 60, 90 and 120 min of extraction under infrared lamp heating. The starting cannabis material contained 10.63% ± 1.46% (w/w) of ∆^9^-THCA, 1.04% ± 0.05% (w/w) of ∆^9^-THC and 0.31% ± 0.01% (w/w) of CBN. Longer extraction times did not result in higher concentrations of the cannabinoids in the ointment bases (Figure 3). Instead, a decrease in ∆^9^-THCA content was observed during prolonged extraction, particularly in the Vaseline base. However, there was no parallel exponential increase in ∆^9^-THC levels (due to possible and expected decarboxylation) or increase in levels of the ∆^9^-THC degradation product CBN. The CBN quantity was under the limit of quantification of this method (LLOQ, <0.025%) in all olive oil and Vaseline extracts sampled during the monitored time. In other decarboxylation studies (Wang et al., 2016; Moreno et al., 2020), the unexplained disappearance of neutral forms was also observed, suggesting that THC may degrade into compounds other than CBN.

Based on the above results, 30 min was set as a sufficient time for cannabinoid extraction to avoid the problems associated with prolonged heating. Moreover, the heating capacity of the infrared lamp was not effective for cannabis decarboxylation even with an extended extraction time. The reachable melting temperature produced by the lamp was 80°C (manufacturer’s information). Therefore, the plant material had to be decarboxylated separately prior to the extraction process.

To increase the extraction process yield, cannabinoid re-extraction from the plant material was also examined for decarboxylated chemotype I cannabis in olive oil and Vaseline. After the first 30 min extraction, the POB was filtered and the remaining plant material on the filter (cotton gauze) was transferred into a new beaker. It was refilled to 30 g with fresh POB, followed by a second 30 min extraction and filtration. The ∆^9^-THC content in both olive oil and Vaseline extracts was less than the LLOQ of the analytical method (<0.025%) after the second extraction. Thus, re-extraction is highly disadvantageous and exhaustive cannabinoid extraction is achieved already in the first step.

The extraction yield of ∆^9^-THC into Vaseline was compared with that obtained using the traditional procedure for cannabis ointment preparation utilized by medical facilities in Czechia. The procedure started with standard decarboxylation at 120°C for 30 min. Then, the blend was mixed with Vaseline and heated at 70°C for 120 min in a hot-air dryer. The extract was placed in a dark, cold place (refrigerator, 7°C) for 2–4 days. Afterwards, the heating process was repeated using the same conditions and the sample was left to rest for 7 days, followed by heating under an infrared lamp to liquefy the ointment and filtration through a cotton gauze. The extract was adjusted to the required weight with the extra Vaseline and thoroughly homogenized. This process was at least ten times longer in comparison with the infrared lamp heat-induced extraction method proposed in this work. However, the extraction yields were comparable for both methods: 0.16% ± 0.00% and 0.14% ± 0.01% (w/w) of ∆^9^-THC for the infrared lamp extraction protocol and traditional procedure, respectively.

Alcohol solvents 96% ethanol and isopropanol were also tested for their extraction capability for cannabinoids. Two extraction procedures were examined: maceration of homogenized plant material in solvent with occasional stirring and sonication in an ultrasonic bath (both with an extraction time of 30 min at laboratory temperature). The ∆^9^-THC extraction yields for both alcohols reached almost 100% in the preparation of 5% cannabis extracts (w/v) (Table 4). In addition, the yields with only random stirring were comparable to sonication extraction.

**TABLE 4:** ∆^9^-THC concentration (mg/mL) and extraction efficiency in 96% ethanol and isopropanol after preparation of 5% cannabis extract (w/v). Mean ± SD (*n* = 3).

Solvent	Type of extraction	∆^9^-THC concentration (mg/mL)	Extraction efficiency (%)
Ethanol 96%	Stirring	2.682 ± 0.007	94
	Sonication	2.779 ± 0.088	97
Isopropanol	Stirring	2.743 ± 0.030	96
	Sonication	2.785 ± 0.045	98

### 3.3 Chemotype extraction

**FIGURE 4:**
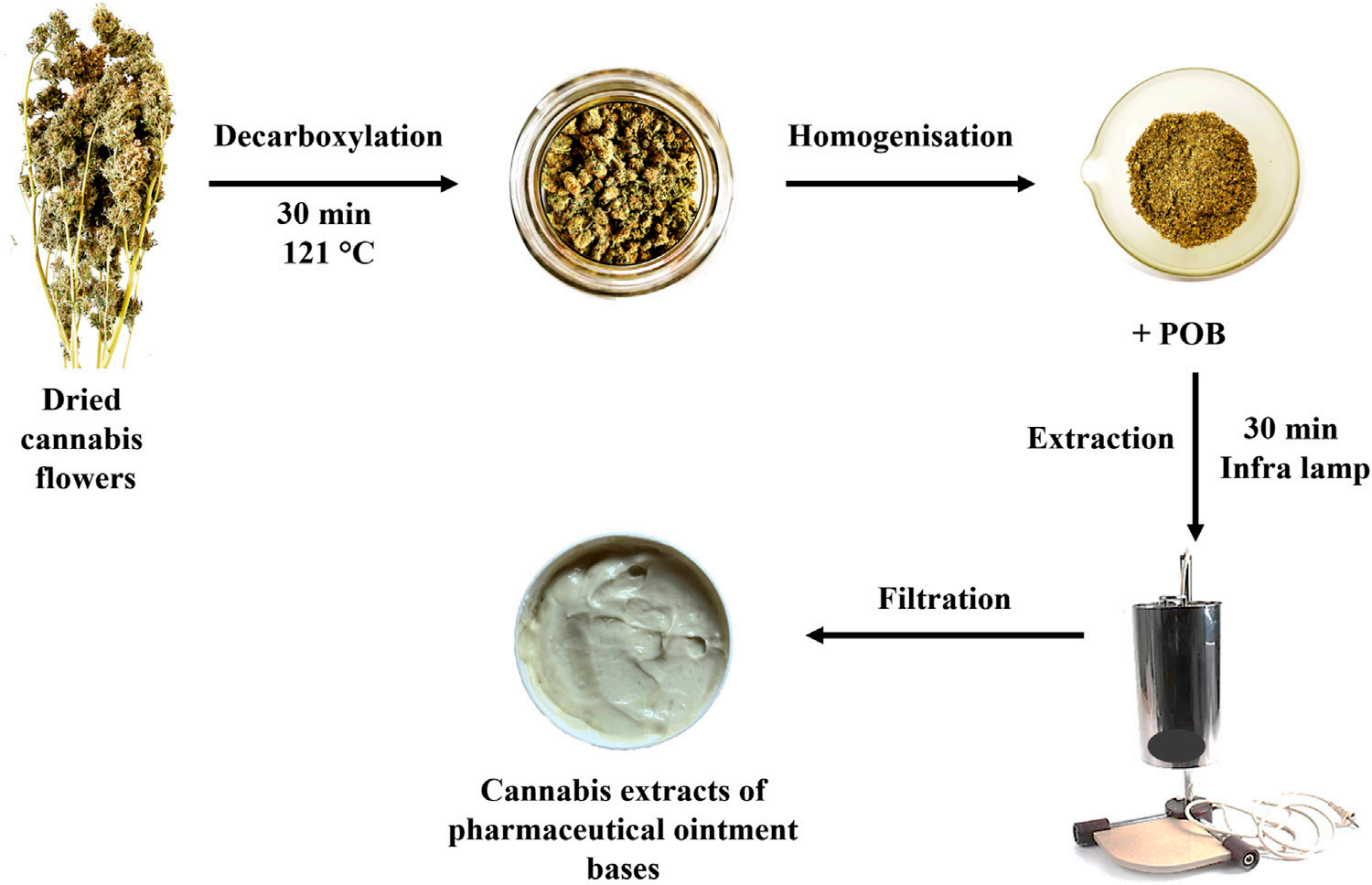
Preparation procedure of cannabis POB extracts proposed in this work.

Six POBs with various physical and chemical properties were selected for cannabinoid extraction from three different chemotypes of *C. sativa*. *Olivae oleum* was chosen as a representative liquid and lipophilic POB. Notably, food-grade olive oil containing cannabinoids could be administered orally. *Vaselinum album* is a readily available stiff hydrophobic ointment base. Synderman (SydoFarm^®^) is a nonaqueous POB composed of Vaseline, paraffin and wool wax (also called *adeps lanae* or lanolin). When mixed with water or other hydrophilic liquid, it generates a hydrophobic cream. AquaNeoFarm^®^ *unguentum* is a nonaqueous POB. In contrast to SydoFarm^®^, it produces a hydrophilic cream when mixed with water or hydrophilic liquid. It is compatible with a wide range of lipophilic substances. Ambiderman (AmiFarm^®^) is a hydrophilic cream suitable for hydrophilic substance incorporation. Its stability depends on the drug concentration and final pH. Pentravan^®^ (penetration enhancing vanishing cream) is an oil-in-water-type cream that allows transdermal drug administration as it penetrates through skin structures into the bloodstream due to its liposomal matrix. The procedure used to prepare cannabis POB extracts (5% (w/w)) is illustrated in Figure 4.

**FIGURE 5:**
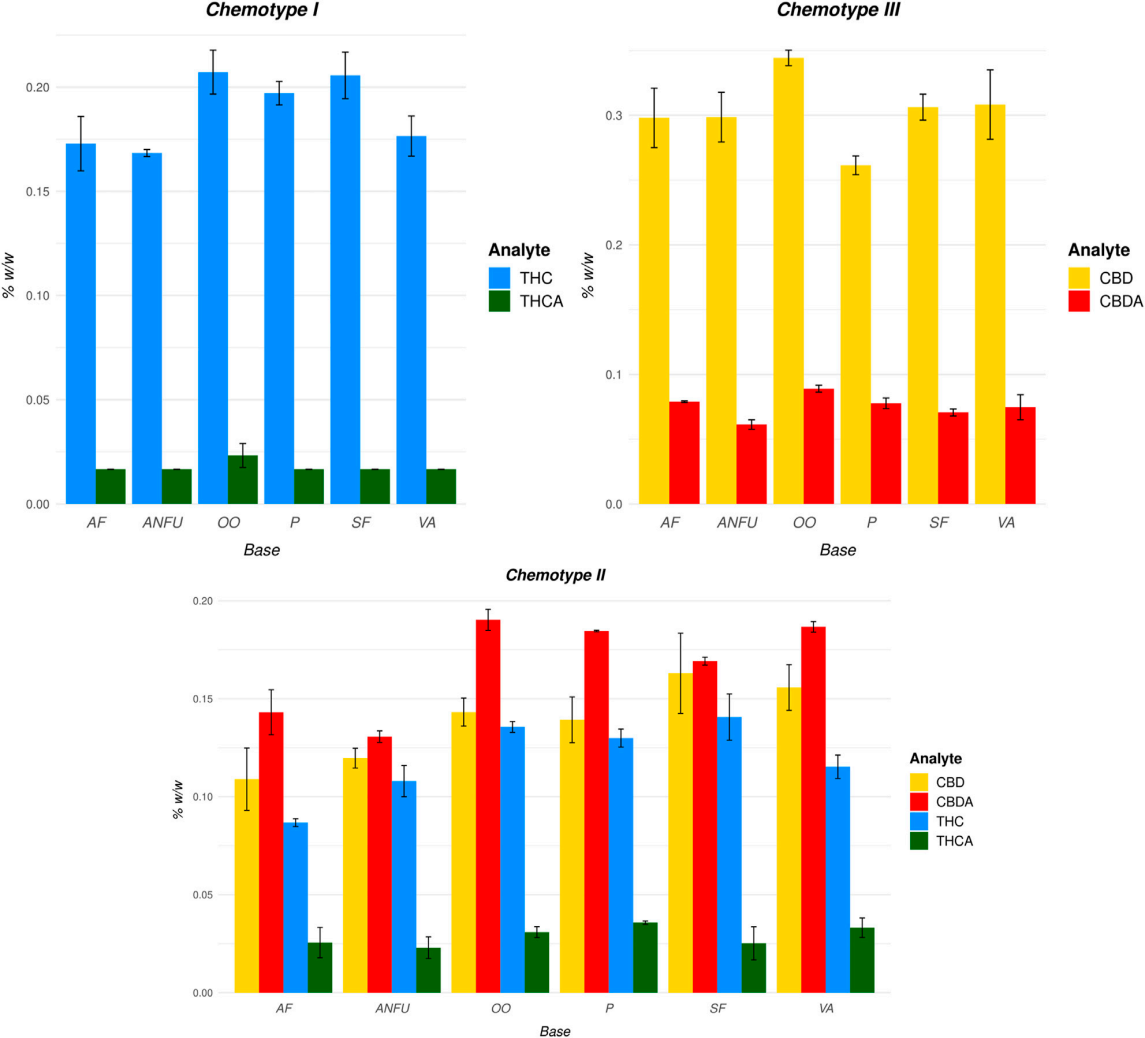
Cannabinoid content (%, w/w) from extracted, decarboxylated cannabis inflorescences of chemotype I (high-THC), chemotype II (THC + CBD), and chemotype III (high-CBD) into POBs. Graphs represent cannabinoids’ mean value and standard deviations of three replicates measured in week 0. In the case of concentrations lower than LLOQ, calculations were performed with 2/3 LLOQ values. AF, AmiFarm^®^; ANFU, AquaNeoFarm^®^ *unguentum*; OO, *olivae oleum*; P, Pentravan; SF, SydoFarm^®^; VA, *vaselinum album*.

All tested vehicles showed extractive capability for acidic and neutral cannabinoids with comparable yields (Figure 5). The highest content of extracted cannabinoids was found to be in the olive oil samples for all chemotypes. Conversely, the lowest amounts were observed in AmiFarm^®^, AquaNeoFarm^®^ *unguentum* and, in the case of chemotype III, in Pentravan^®^ extracts. However, the differences in total cannabinoid content in the POBs extracts were not significant.

**FIGURE 6:**
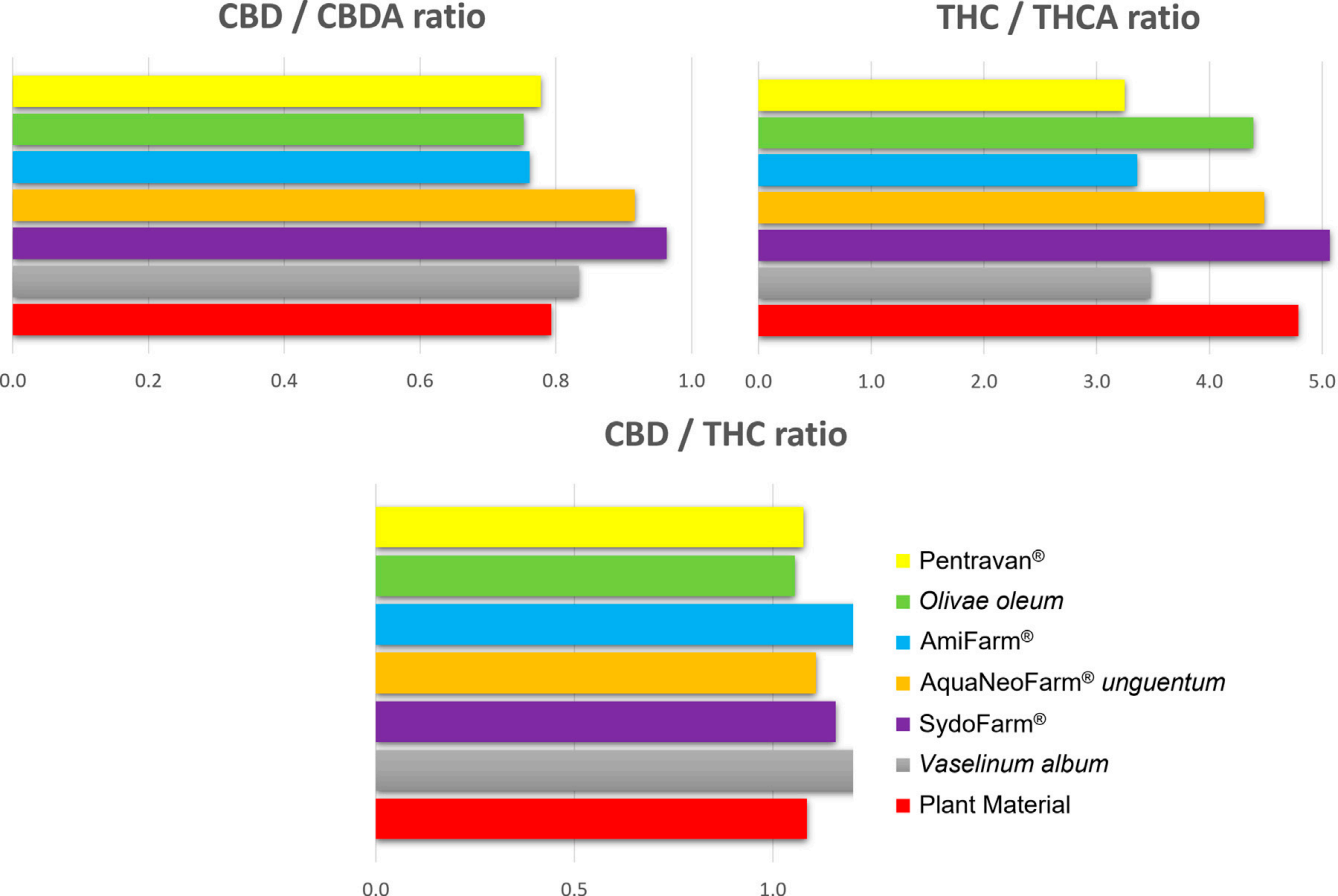
Comparison of the ratios of major cannabinoids extracted into different pharmaceutical bases and in the original decarboxylated plant material (cannabis Chemotype II).

In the chemotype II extracts, the affinity of individual POBs for cannabinoids was evaluated as this chemotype contained both major cannabinoids (CBD, ∆^9^-THC) and the concentration levels of acidic and neutral CBD were similar. In all tested POBs, the ratio of extracted CBD to CBDA was similar to that in the original (decarboxylated) plant material (Figure 6). This indicates that the bases did not preferentially extract either the neutral or acidic cannabinoids. In addition, the ratio of CBD to ∆^9^-THC content was similar to the chemical composition of the cannabis. Therefore, the POBs were capable of extracting both predominant cannabinoids comparatively.

### 3.4 Stability test

**FIGURE 7:**
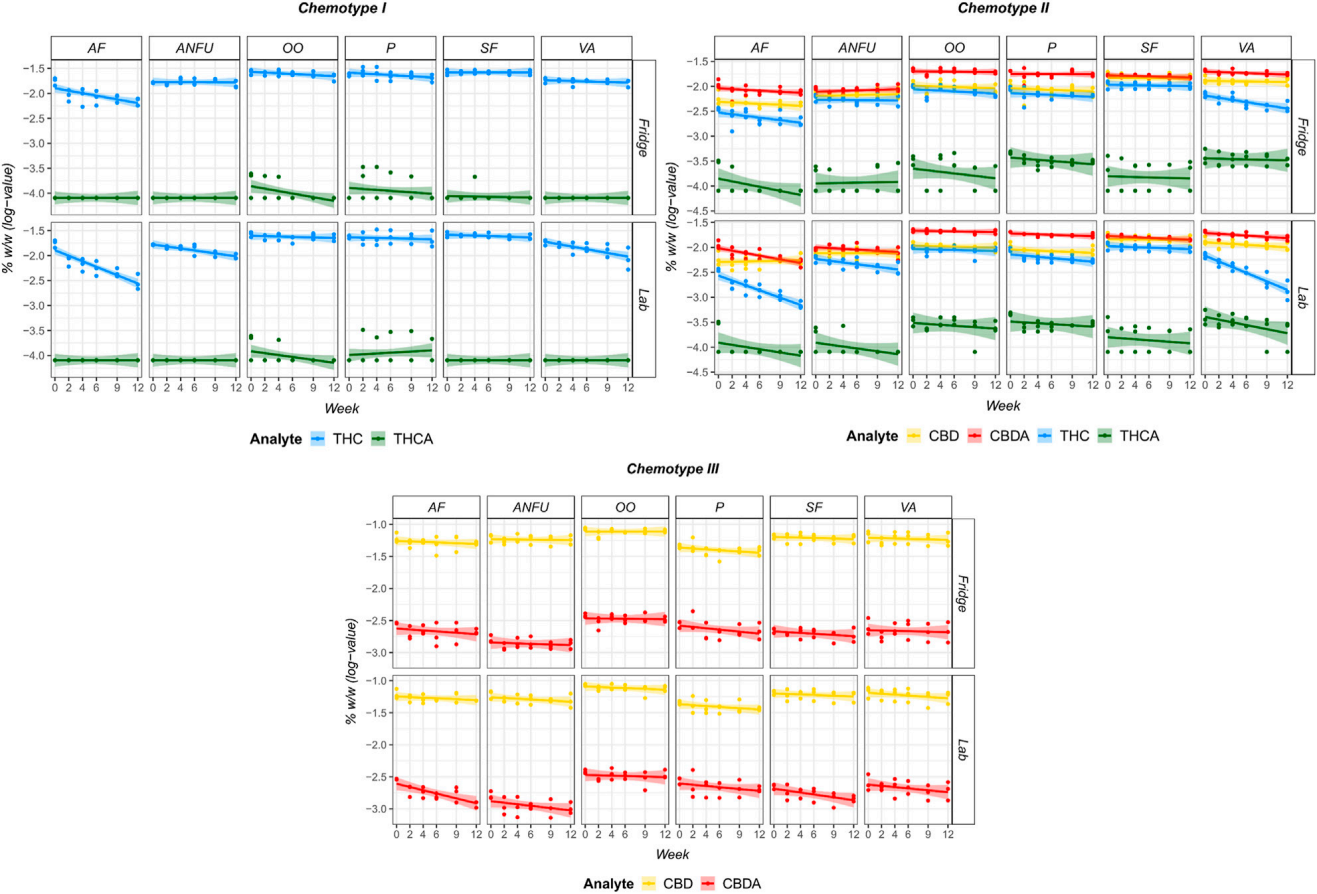
Changes in cannabinoid content (%, w/w) in POB extracts of chemotype I, II, and III inflorescences over the monitoring period of 12 weeks stored at laboratory temperature (LAB) or in a refrigerator at 7°C (FRIDGE). Graphs display the actual values (in log-scale) and the fitted regression line (with 99% confidence interval). In the case of concentrations lower than LLOQ, calculations were performed with 2/3 LLOQ values. AF, AmiFarm^®^; ANFU, AquaNeoFarm^®^ *unguentum*; OO, *olivae oleum*; P, Pentravan^®^; SF, SydoFarm^®^; VA, *vaselinum album*.

Cannabinoid concentrations in the POBs extracts were monitored during 3 months with a special focus on the first month to verify the shelf life as medical cannabis is prescribed monthly. Changes in the cannabinoid concentrations are shown in Figure 7, and the estimated percentage losses per week are presented in Table 5.

**TABLE 5:** Estimated percentage changes (%; with 99% confidence interval) in the total cannabinoid content per week. Statistically significant changes are marked in yellow. The last column displaces the coefficients of the determination (R^2^) for the respective models.

Chemotype	Cannabinoid	Temperature/Base	AF	ANFU	OO	P	SF	VA	*R* ^2^
**I**	**∆^9^-THC**	**Refrigerated**	**−2.5** (−3.7, −1.2)	**−0.1** (−1.3, −1.2)	**−0.7** (−2.0, 0.6)	**−0.8** (−2.0, 0.5)	**−0.0** (−1.3, 1.3)	**−0.4** (−1.7, 0.9)	0.8674
		**Lab**	**−5.4** (−6.6, −4.2)	**−2.0** (−3.2, −0.7)	**−0.4** (−1.6, 0.9)	**−0.3** (−1.6, 1.0)	**−0.4** (−1.7, 0.8)	**−2.5** (−3.7, −1.2)	
**II**	**CBD**	**Refrigerated**	**−0.7** (−2.0, 0.7)	**0.3** (−1.1, 1.6)	**−0.4** (−1.8, 0.9)	**−0.5** (−1.8, 0.8)	**−0.2** (−1.5, 1.2)	**−0.2** (−1.5, 1.2)	0.7784
		**Lab**	**0.3** (−1.0, 1.7)	**−0.0** (−1.3, 1.3)	**−0.3** (−1.6, 1.0)	**−0.6** (−1.9, 0.8)	**−0.2** (−1.5, 1.2)	**−0.8** (−2.1, 0.5)	
	**CBDA**	**Refrigerated**	**−0.8** (−1.7, 0.2)	**0.4** (−0.5, 1.4)	**−0.1** (−1.0, 0.8)	**−0.0** (−1.0, 0.9)	**−0.4** (−1.3, 0.6)	**−0.5** (−1.4, 0.4)	0.8949
		**Lab**	**−2.5** (−3.4, −1.5)	**−0.8** (−1.7, 0.1)	**−0.2** (−1.2, 0.7)	**−0.5** (−1.4, 0.5)	**−0.6** (−1.5, 0.3)	**−0.9** (−1.8, 0.1)	
	**∆^9^-THC**	**Refrigerated**	**−1.7** (−3.0, −0.3)	**−0.1** (−1.5, 1.3)	**−0.7** (−2.1, 0.7)	**−0.7** (−2.0, 0.7)	**−0.2** (−1.6, 1.2)	**−2.1** (−3.5, −0.8)	0.9042
		**Lab**	**−4.8** (−6.1, −3.5)	**−1.7** (−3.1, −0.4)	**−0.3** (−1.7, 1.1)	**−1.2** (−2.6, 0.2)	**−0.5** (−1.9, 0.9)	**−5.6** (−6.9, −4.2)	
	**∆^9^-THCA**	**Refrigerated**	**−2.7** (−5.6, 0.3)	**0.2** (−2.8, 3.3)	**−1.6** (−4.6, 1.4)	**−1.1** (−4.1, 2.0)	**−0.4** (−3.4, 2.7)	**−0.3** (−3.3, 2.8)	0.5724
		**Lab**	**−2.1** (−5.1, 0.9)	**−1.9** (−4.8, 1.1)	**−0.9** (−3.9, 2.1)	**−0.9** (−3.8, 2.2)	**−1.0** (−4.0, 2.1)	**−2.7** (−5.6, 0.3)	
**III**	**CBD**	**Refrigerated**	**−0.4** (−1.5, 0.7)	**−0.1** (−1.2, 0.9)	**0.0** (−1.1, 1.1)	**−0.7** (−1.7, 0.4)	**−0.3** (−1.3, 0.8)	**−0.3** (−1.3, 0.8)	0.6404
		**Lab**	**−0.5** (−1.5, 0.6)	**−0.6** (−1.6, 0.5)	**−0.4** (−1.5, 0.7)	**−0.7** (−1.7, 0.4)	**−0.4** (−1.5, 0.7)	**−0.7** (−1.8, 0.4)	
	**CBDA**	**Refrigerated**	**−0.7** (−2.2, 0.8)	**−0.3** (−1.8, 1.1)	**−0.1** (−1.6, 1.4)	**−1.0** (−2.5, 0.4)	**−0.6** (−2.1, 0.9)	**−0.3** (−1.7, 1.2)	0.6848
		**Lab**	**−2.5** (−3.9, −1.1)	**−1.2** (−2.6, 0.3)	**−0.3** (−1.8, 1.2)	**−1.0** (−2.4, 0.5)	**−1.5** (−2.9, −0.0)	**−1.0** (−2.4, 0.5)	

Lab, laboratory temperature; ∆^9^-THC, (-)-*trans*-∆^9^-tetrahydrocannabinol; ∆^9^-THCA, (-)-*tran*s-∆^9^-tetrahydrocannabinolic acid; CBD, cannabidiol; CBDA, cannabidiolic acid; AF, AmiFarm^®^; ANFU, AquaNeoFarm^®^ *unguentum*; OO, *olivae oleum*; P, Pentravan^®^; SF, SydoFarm^®^; VA, *vaselinum album*.

A large decrease in THC concentration was observed in extracts of chemotype I to AmiFarm^®^, AquaNeoFarm^®^ *unguentum* and *vaselinum album* stored at laboratory temperature (Figure 7). The initial concentrations of THC in AmiFarm^®^ and *vaselinum album* were 0.17% ± 0.01% and 0.18% ± 0.01% (w/w), respectively. After 1 month of storage at laboratory temperature, the concentrations decreased to 0.11% ± 0.01% and 0.16% ± 0.02% (w/w), and after 12 weeks, to 0.08% ± 0.01% and 0.13% ± 0.03% (w/w) in AmiFarm^®^ and *vaselinum album*, respectively. According to the estimated relative changes (Table 5), the loss of THC content per week in extracts stored at laboratory temperature was 5.4%, 2% and 2.5% in AmiFarm^®^, AquaNeoFarm^®^ *unguentum* and Vaseline, respectively. In the case of chemotype I extracts stored at low temperature, a significant decrease in THC content was observed only in the AmiFarm^®^ samples.

A similar trend of THC degradation was also shown in the chemotype II extracts. Moreover, a significant change in THC content was observed in Vaseline extracts stored in a refrigerator. Furthermore, CBDA showed a tendency to decompose by about 2.5% per week in AmiFarm^®^ extracts stored at laboratory temperature (Table 5). Owing to the same temperature conditions, significant CBDA decomposition was expected in the chemotype III extracts of AmiFarm^®^ and SydoFarm^®^. However, CBD concentrations were relatively stable in all extracts and under all storage conditions (Figure 7; Table 5).

The different physical and chemical properties of the POBs had an impact on the final extract texture and structure during preparation and storage. The AmiFarm^®^ extracts could not be easily filtered and particles of the plant material were present in the ointment, which may have affected the final cannabinoid content. In the case of Pentravan^®^, its structure disintegrated and an oily layer formed on the surface of the prepared ointments. The rest of the POBs was easier to filter as they liquefied smoothly with heat and the extracts were relatively pure, with only fine sediment appearing at the bottom of the tubes during storage. Nevertheless, it is not recommended to store pharmaceutical-grade olive oil extracts in the refrigerator because at low temperatures, they change their structure and partially solidify. Moreover, following the extraction process and filtration, the extracts were adjusted to the original weight by adding a fresh POB. In the case of the cream bases (AmiFarm^®^, Pentravan^®^), up to half of the base’s original weight was lost during the filtration process. This could require consumption of more POB material, making the final product more expensive.

To conclude, *olivae oleum* and Synderman (SydoFarm^®^) extracts showed the best stability of all tested POBs during the monitored period. Therefore, they may be promising vehicles for the preparation of pharmaceutical dosage forms from cannabis, offering long shelf life concerning the cannabinoid concentration.

## 4 Discussion

In this work, two different UHPLC-UV methods were applied for cannabinoid analysis based on national pharmacopeial methods (OMC, USP). They differed in the chromatographic conditions, particularly column properties and mobile phases (described in section 2. *Material and Methods*). Use of these methods resulted in different cannabinoid separations, and therefore possible quantification of analytes. The main disadvantage of using the OMC chromatographic conditions (Method I) was the coelution of CBG and CBGA. Thus, their concentrations in the sample could only be expressed as the sum. However, this method succeeded in separating CBNA and ∆^8^-THC, which was not possible by the USP method (Method II). CBNA monitoring was necessary for the ointment extracts due to the possible degradation of ∆^9^-THCA during storage. Therefore, Method I was preferred for the extract analysis. On the other hand, CBG and CBGA resolution was essential for detailed chemical characterization of cannabis chemotype inflorescences, which was achievable by Method II. This dual approach allowed selection of the best method according to the sample properties and the required compound quantification.

Analysis of decarboxylation was not the primary objective of this paper. However, it became a stimulus for discussion due to many inconsistencies between previous studies. Baratta et al. (2019) tested the time for cannabinoid extraction (30, 60, 120 min) into olive oil and the influence of two decarboxylation processes of the raw material in comparison with no preheating. For warm extraction, they used a boiling water bath. Based on their results, an extended time of extraction was deemed not necessary (120 min) and they recommended an optimal extraction time of 60 min under the given conditions. In the present study, a 30 min extraction time under an infrared lamp was found to be sufficient for olive oil and Vaseline, with no need for lengthy preparation. Moreover, in Baratta et al.’s study, after 120 min heating of raw (non-decarboxylated) cannabis in the water bath, the acidic forms still prevailed over the decarboxylated ones, similarly to the current study. Comparing two different decarboxylation conditions, higher levels of decarboxylated forms were achieved when conditions of 140°C for 30 min were applied compared to a lower temperature for a longer period (115°C for 40 min) (Baratta et al., 2019). Romano and Hazekamp (2013) heated chemotype I cannabis at 145°C for 30 min, which resulted in complete decarboxylation of ∆^9^-THCA. However, it should be noted that such a high temperature was probably beyond the evaporation point of ∆^9^-THC (Wang et al., 2016). In the present work, preheating the raw material at 121°C for 30 min followed common practice in Czech pharmacies. In addition, Baratta et al. (2019) recommended homogenization and spreading of the material in a thin layer for uniform heating (maximum 5 mm, preferably 1–2 mm), as well as the Italian monograph (maximum thickness of the cannabis layer 1 cm) (Società Italiana Farmacisti Preparatori, 2016). In the present work, trimmed inflorescences were heated without pre-homogenization. These findings indicate that temperature and pre-homogenization of the plant material, with emphasis on layer thickness, are key aspects for effective decarboxylation.

In light of the above observations, the decarboxylation process in cannabis flowers should be optimized and studied to achieve higher yields of the decarboxylated forms. The process of decarboxylation optimization could be problematic in the case of chemotype II samples since they contain both major cannabinoids. ∆^9^-THCA decarboxylates more rapidly than CBDA, potentially increasing levels of CBN, which is restricted in medical cannabis products. Previous kinetic studies reported unexplained losses of reactants or products during heating in the case of CBDA decarboxylation (Wang et al., 2016; Citti et al., 2018; Moreno et al., 2020). Despite this, acidic cannabinoids may also be desirable compounds in dermatological applications due to cannabinoid synergistic interactions. Indeed, the entourage effect has been extensively discussed and studied (Russo, 2011; Koltai and Namdar, 2020),. For example, cannabinoid-cannabinoid synergy resulted in an increased plasma concentration of CBDA when the extract was administered orally in comparison with single molecule administration (Anderson et al., 2021).

To the best of our knowledge, our study is the first to report the extractive capabilities of a wide range of POBs for phytocannabinoids. Casiraghi et al. (2020) studied the influence of vehicle-related aspects on the skin permeation process. However, although they tested different formulations (liquids, semisolids), they only investigated pure CBD and not extracts or other cannabinoids. Majority of the studies have aimed to the development of innovative methods for the preparation of cannabis galenic oil formulations (Società Italiana Farmacisti Preparatori, 2016; Casiraghi et al., 2018; Baratta et al., 2019; Ternelli et al., 2020; Baratta et al., 2021) and their quality assessment (Bettiol et al., 2019; Dei Cas et al., 2020), or cannabinoid extraction using lipid-based vehicles (Aguirre et al., 2023). Nevertheless, Aguirre et al.’s work did not focus on stability testing of the prepared formulations. In the present study, the three most commonly used medical cannabis chemotypes were investigated. All tested POBs showed, to some extent, extractive capabilities for cannabinoids. The highest extraction efficiencies (EE, %) were observed for *olivae oleum* and Synderman^®^, whereas the lowest were for cream bases. However, the lower content of cannabinoids in other POBs extracts may have been due to differences in the filtration process, i.e., wetting of the cotton gauze and glassware by the POBs.

The optimal time for quantitative cannabinoid extraction induced by heat was shown to be 30 min since a decrease in cannabinoid content (specifically ∆^9^-THCA) in *olivae oleum* and *vaselinum album* was observed at longer time (up to 120 min). Furthermore, re-extraction of the plant material used in the first 30 min extraction was examined. As a result, the ∆^9^-THC levels were below the LLOQ (<0.025%). Thus, adding more ointment base did not increase the cannabinoid extractability into *olivae oleum* or *vaselinum album*. Levels of CBN (degradation product of THC), which are limited by implementing decree (Ministry of Health and Ministry of Agriculture, 2015), were below the method LLOQ in all extracts, consistent with the findings of a previous study of olive oil extracts (Citti et al., 2016) and Baratta et al. (2019), where CBN was present in minimal amounts in the oils.

The Italian monograph stipulates a 1:10 ratio (1 g of cannabis/10 mL of olive oil) for the preparation of cannabis oil extracts in pharmacies (Società Italiana Farmacisti Preparatori, 2016), which corresponds to preparation of a 10% extract (w/v). By adhering to this ratio, the extraction rate of total CBD (sum of CBDA and CBD) was about 78% in olive oil (Citti et al., 2016). In the present work, 5% extracts (w/w) with approximately 70% of the extractive yield of ∆^9^-THC and CBD in olive oil extracts were prepared since this concentration is generally formulated in Czech pharmacy practice. Similarly, Citti et al. (2016) concluded that the stability of cannabinoids in solution (olive oil and 96% ethanol) was dependent on storage temperature (8°C *versus* 25°C). Furthermore, they observed higher decomposition of ∆^9^-THC compared to other cannabinoids, matching our obtained data.

In the present study, cannabinoid extraction from homogenized plant material into alcohol solvents (96% ethanol and isopropanol) at laboratory temperature was also investigated. A procedure that could be routinely performed in a pharmacy with basic equipment was compared with ultra-sonic extraction, which generally results in higher yields. However, the ∆^9^-THC recoveries were comparable for both preparation methods and extraction solvents. The extraction efficiency for cannabinoids was almost 100% for both tested alcohols. The main advantage of our method is that there is no high loss of biologically active molecules during the extraction and filtration process. Furthermore, cannabis tinctures could be easily incorporated into suitable alcohol-tolerant ointment bases and titrated to the required cannabinoid concentration. In this case, an isopropanol tincture would be preferable as it is less drying to the skin compared to ethanol. On the other hand, isopropanol is only applied externally, whereas ethanol tinctures and extracts could also be used orally or via inhaler. Moreover, propylene glycol, which can also be used for oral drug administration, showed relatively high ∆^9^-THC extraction efficiency (70%) (Table 3). However, Citti et al. (2016) observed rapid decomposition of cannabinoids in 96% ethanolic extract during storage. Therefore, the tinctures should be mixed with an appropriate POB immediately after preparation.

During preparation and storage, changes in the structure of some POBs were observed, especially for AmiFarm^®^, Pentravan^®^ and AquaNeoFarm^®^ *unguentum*. Therefore, these ointment bases are not recommended for the preparation of extracts by the protocol proposed here. Besides this experiment, in our laboratory, the incorporation of cannabis extracts (supercritical fluid CO_2_ extract (SFE), evaporated alcohol extract) into Ambiderman (AmiFarm^®^) has been tested. The result was a homogeneous cream with no observed disintegration or change in structure even during storage for months at low temperatures (refrigerator). However, the stability of cannabinoids in the ointment was not monitored. This cream base contains a water phase consisting of carbomer gel (polyacrylic acid) which could affect the stability of active ingredients. In the current study, a rapid decrease in ∆^9^-THC and acidic cannabinoid forms was observed in AmiFarm^®^ extracts stored at laboratory temperature or in a refrigerator. Since the Ambiderman cream contained the highest amount of water of all the tested POBs (70% of water), a high water content could have a negative impact on THC stability via hydrolysis. Moreover, the stability of galenic preparations of Ambiderman depends on the concentration of incorporated compounds and resulting pH. Conversely, CBD was stable in the cream extracts at both temperatures during the monitoring time. These observations suggest that cannabinoids are variably stable in ointment bases depending on their physical and chemical properties.

The Pentravan^®^ cream was expected to be a promising ointment base since it penetrates through skin layers due to its chemical composition and could be used for transdermal administration of cannabinoids into the bloodstream, which is desirable for patients who cannot inhale or take cannabis orally. However, its structure disintegrated during the heated extraction and storage. Furthermore, the cream formulation was not easy to filter through cotton gauze to remove the plant particles. In future work, incorporation of a cannabis extract (SFE, ethanolic, oil) into the cream could be tested with monitoring of its stability.

The law novelization and recent extension of the Czech implementing decree (Ministry of Health and Ministry of Agriculture, 2015; Ministry of Health and Ministry of Agriculture, 2022) have allowed prescription of medical cannabis extracts for treatment, which could potentially be used for the preparation of galenic formulations. This could facilitate the preparation of ointments because with a known concentration of substances in the extract, it would be easier to dilute the cannabinoid content and standardize the final product. Besides dermatological applications, some algesiologists have expressed interest in the transdermal administration of medical cannabis for pain therapy. Nevertheless, extracts are not a commonly available drug form in Czech medical practice. Moreover, physicians and patients have experienced a shortage of medical cannabis flowers in the Czech market in the past. A similar situation might also be expected for extracts in the future. Therefore, it is essential to have a protocol for the preparation of cannabis-based galenic formulations from raw plant material (inflorescence) that could be easily employed by pharmacists. In general, the usual preparation procedures in pharmacies are unnecessarily time-exhaustive. The preparation procedure proposed in this work is simple and less time-consuming and could be applied routinely even in the absence of medical cannabis extracts.

This research paper is primarily focused on the phytocannabinoid extraction and stability in cannabis-based galenic formulations for topical application. We foresee an opportunity for future related studies that should be dedicated to terpenoid evaluation as these compounds are also the main active constituents of cannabis with pharmacological effects including antimicrobial or anti-inflammatory activities (Hanuš and Hod, 2020). Moreover, the presented results on the stability of the active compounds may be beneficial for the pharmaceutical practice, since medical cannabis is prescribed with a shelf life of 1 month with possible prolongation depending on the physical and chemical properties of the prepared galenic formulations (Czech Pharmacopoeia, 2017-Appendix, 2022). However, the assays described in Pharmacopoeia should be performed to assess the quality of the prepared formulations, including microbiological safety.

## 5 Conclusion

The present study demonstrated the extractive capability of various pharmaceutical ointment bases for phytocannabinoids extracted from different *Cannabis sativa* L. chemotypes. The prepared extracts showed variable stability during storage depending on the physical and chemical properties of the pharmaceutical bases and compounds of interest. Furthermore, a simple and rapid protocol for the preparation of cannabis-based galenic formulations intended for dermatological applications was proposed since phytocannabinoids have been shown to be effective treatments for many skin disorders through regulation of the endocannabinoid system. However, at present, a standardized procedure is lacking in Czech medical practice and pharmacies.

Our results showed that olive oil and Synderman bases exhibited the highest cannabinoid extraction efficiencies (approximately 70%) and best storage stability in terms of the content of the monitored compounds. On the other hand, the cream bases were the least stable and problematic for extract preparation via the proposed protocol. Regarding cannabinoid stability, ∆^9^-tetrahydrocannabinol was less stable compared to cannabidiol and decomposed rapidly in certain bases, especially when stored at laboratory temperature.

In conclusion, the findings of this study provide valuable insights into appropriate selection of carriers for the preparation of phytocannabinoid-rich topical galenic formulations. Overall, the study could support the practical incorporation of medical cannabis into dermatological treatment.

## Data Availability

The raw data supporting the conclusion of this article will be made available by the authors, without undue reservation.
